# Feasibility of sentinel lymph node biopsy omission after integration of ^18^F-FDG dedicated lymph node PET in early breast cancer: a prospective phase II trial

**DOI:** 10.20892/j.issn.2095-3941.2022.0085

**Published:** 2022-07-21

**Authors:** Junjie Li, Jingyi Cheng, Guangyu Liu, Yifeng Hou, Genghong Di, Benglong Yang, Yizhou Jiang, Liang Huang, Feilin Qu, Sheng Chen, Yan Wang, Keda Yu, Zhimin Shao

**Affiliations:** 1 Department of Breast Surgery, Fudan University Shanghai Cancer Center, and Department of Oncology, Shanghai Medical College, Fudan University, Shanghai 200032, China; 2 Key Laboratory of Breast Cancer in Shanghai, Fudan University Shanghai Cancer Center, Fudan University, Shanghai 200032, China; 3 Department of Nuclear Medicine, Fudan University Shanghai Cancer Center, Shanghai 200032, China

**Keywords:** Breast cancer, sentinel lymph node biopsy, ^18^F-fluorodeoxyglucose, LymphPET, negative predictive value

## Abstract

**Objective:**

Sentinel lymph node biopsy (SLNB) is currently the standard of care in clinically node negative (cN0) breast cancer. The present study aimed to evaluate the negative predictive value (NPV) of ^18^F-FDG dedicated lymph node positron emission tomography (LymphPET) in cN0 patients.

**Methods:**

This was a prospective phase II trial divided into 2 stages (NCT04072653). In the first stage, cN0 patients underwent axillary LymphPET followed by SLNB. In the second stage, SLNB was omitted in patients with a negative preoperative axillary assessment after integration of LymphPET. Here, we report the results of the first stage. The primary outcome was the NPV of LymphPET to detect macrometastasis of lymph nodes (LN-macro).

**Results:**

A total of 189 patients with invasive breast cancer underwent LymphPET followed by surgery with definitive pathological reports. Forty patients had LN-macro, and 16 patients had only lymph node micrometastasis. Of the 131 patients with a negative LymphPET result, 16 patients had LN-macro, and the NPV was 87.8%. After combined axillary imaging evaluation with ultrasound and LymphPET, 100 patients were found to be both LymphPET and ultrasound negative, 9 patients had LN-macro, and the NPV was 91%.

**Conclusions:**

LymphPET can be used to screen patients to potentially avoid SLNB, with an NPV > 90%. The second stage of the SOAPET trial is ongoing to confirm the safety of omission of SLNB according to preoperational axillary evaluation integrating LymphPET.

## Introduction

Sentinel lymph node biopsy (SLNB) has replaced axillary dissection (AD) as the standard surgical procedure, because of its similar diagnostic accuracy and prognosis, but lower surgical morbidity^1–3^. However, surgical morbidities still exist in patients with SLNB, one-quarter of which occur in the early postoperative period^4^ and 4% of which occur in the late period^5^. A recent review of 7 trials involving 9,426 participants has found that the surgical complications of SLNB include lymphedema in 4.8%, subjective arm movement impairment in 4%, paresthesia in 34%, pain in 8.6%, and numbness in 18.5% of patients^6^.

With the wider adoption of mammography screening, the early diagnosis rate of breast cancer has increased^7^, and approximately two-thirds of clinical node negative (cN0) patients are pathology node negative after SLNB^3^. Therefore, investigating the omission of SLNB in cN0 patients to avoid further physical and emotional distress is of considerable interest. To date, the accuracy of physical examination combined with ultrasound, magnetic resonance imaging (MRI), and even ^18^F-fluorodeoxyglucose (^18^F-FDG) positron emission tomography (PET) for preoperative axillary evaluation has not been satisfactory [negative predictive value (NPV) < 85%]^8,9^. Increasing the NPV of axillary assessments to > 90% in preoperative nodal staging is a key unmet need for clinicians determining whether to omit SLNB.

According to our previous study, a novel high-resolution dedicated axillary lymph node (LN) PET (LymphPET) method can be used to identify and recognize more indolent axillary LNs in breast cancer, given its greater sensitivity than other methods and its NPV of 90%^10^. Hence, we conducted a prospective phase II study to investigate the NPV of LymphPET and to verify whether SLNB might be omitted in cN0 patients (SOAPET, NCT04072653).

## Patients and methods

### Study design and patients

Sentinel node biopsy *vs*. observation after axillary PET (SOAPET) is a prospective, open-label, phase II clinical trial conducted at Fudan University Shanghai Cancer Center. The study was divided into 2 stages. The study protocol is available in the **Data Supplement (online only)**. In the first stage, cN0 patients were identified through clinical examination and underwent axillary imaging evaluation and LymphPET, followed by axillary surgery (SLNB or AD). In the second stage, SLNB was omitted for patients with a negative preoperative axillary assessment after integration of LymphPET (PEMTECHTM, Shanghai, China). Here, we report the results of the first stage.

The eligibility criteria were as follows: women ≥ 18 years old with newly diagnosed, histologically confirmed unilateral invasive breast cancer and a negative axillary physical examination. The key exclusion criteria were the presence of distant metastases; previous neoadjuvant therapy; previous axillary biopsy or axillary surgery 7 days before LymphPET; current pregnancy or lactation; and diabetes mellitus without blood glucose management.

The study protocol was approved by independent ethics committees at Fudan University Shanghai Cancer Center, and the study was conducted in accordance with good clinical practice and the Declaration of Helsinki. Informed consent complying with all applicable laws and regulations concerning the privacy and/or security of personal information was obtained from the participants or their legal guardians before study participation.

### Procedures

Patients with a negative axillary physical examination underwent routine breast and axillary imaging evaluation (all patients underwent axillary ultrasound and LymphPET). The results of axillary ultrasound and LymphPET were recorded. After diagnosis of invasive carcinoma through core needle biopsy in breast lesions, all enrolled patients underwent surgical operation and pathological evaluation of axillary LNs.

#### LymphPET system and examination

The LymphPET device contains movable double-planar confronted detectors with an axillary view and an adjustable distance between the 2 detector plates ranging from 8 to 37 cm. The size of the sensitive detection area is 208 × 208 mm. Both the bilateral breasts and axillary nodes were scanned. To detect the metastatic status of ALNs, the patient’s axilla is positioned in the middle of the bi-planar detectors, and the detectors close to the patient show higher sensitivity. Each square-shaped detector plane is 20 cm × 20 cm and is composed of 16 units of double-sided front-end readout modules that integrate the LYSO crystal arrays and 2 SiPM array frontend electronics in a compact detector module.

Patients fasted at least 6 h before receiving a standardized injection of 4 mCi ^18^F-FDG (injection in the contralateral arm to the breast lesion). Blood glucose levels were required to be less than 10 mmol/L. After a resting period of 60 min to allow for tracer distribution, LymphPET was performed. The acquisition duration was 3 minutes for each region, and both bilateral axillary regions were detected individually. After acquisition, the images were reconstructed with a 3D standard maximum likelihood expectation maximization (MLEM 3D) algorithm and verified immediately.

#### LymphPET image analysis

According to our previous study, to evaluate Lymph-PET images and quantify the single-voxel maximum standard uptake value (SUVmax), we used commercial Medical Image Merge (version 6.5.4; MIM Software Inc., Beachwood, OH, USA), a professional image processing program certified by the U.S. Food & Drug Administration. Two nuclear medicine physicians with 10 years of experience in PET/CT, who were blinded to study-associated information in addition to the laterality of BC, analyzed the images separately. The elliptic-shaped region of interest (ROI) was manually delineated, and ^18^F-FDG uptake (SUVmax) was calculated in the delineated ROI. The highest SUVmax was selected as the study value when multiple LNs were detected and was defined as the maximum single-voxel standardized LUV (maxLUV). ALNs were considered positive under the following 3 conditions: (1) the positive focus was located in the axillary region but not in skin, muscle, or bone; (2) ^18^F-FDG uptake was greater than the reference background (fat tissue); and (3) the physiological lymphatic uptake was excluded, such as a symmetrically bilateral positive focus, like 2 funicular ropes. For quantitative analysis, the minor diameter of the LN was measured, and an elliptical ROI was drawn manually. ^18^F-FDG uptake into this ROI was calculated as the ALN maxLUV. The highest maxLUV was selected as the study value in the event that multiple LNs were detected. Additionally, 3 separate ROIs measuring 1 cm in diameter (fat background) were located at the axillary adipose tissue, and the mean value of these areas was defined as maxLUV fat. Moreover, 3 1-cm diameter ROIs were located at the biceps brachii and ectopectoralis muscles (muscle background), and the mean value was denoted maxLUV muscle. According to our previous study, when the cut-off value of the maxLUV LN was set at 0.27 (as recommended by Youden’s index), the diagnostic sensitivity was 88%, and the NPV reached a maximum of 90%, which was the best cut-off value for identifying the most suitable indicator for detecting ALNs with LymphPET^10^.

#### Ultrasound analysis

Axillary ultrasounds were performed and read by experienced radiologists. Transverse and longitudinal scans were obtained, and the diameter and cortical thickness of the LNs were measured. LNs were evaluated on the basis of their shape, border, and echogenicity.

If no suspicious LNs were detected, the case was defined as US-neg. If abnormalities in the LN hilus, and/or cortex thickening, and/or the length-width ratio suggested a possibility of non-metastasis, the case was defined as US-det. If LNs exhibited one or more of the following characteristics, the case was defined as US-met: cortical thickening or eccentric cortical lobulation with obliteration of echogenic hilum, irregular shape length-width ratio 1:1, or loss of fatty hilum.

### Surgical procedure and pathological evaluation of LNs

SLNB was performed in cN0 patients. Patients with ultrasound-detected LNs underwent ultrasound-guided fine-needle aspiration; if the results were negative, SLNB was routinely performed.

The SLN was identified with blue dye and/or radiocolloid. SLNs were defined as any blue-stained node, any node with a blue-stained lymphatic channel directly leading to it, any node with radioactive counts ≥ 10%, or any pathologically palpable nodes. Touch imprint cytology was routinely used for every SLN harvested. Additionally, slices were formalin fixed and paraffin-embedded for further evaluation. Serial sectioning with HE staining was performed. The pathological results were classified as macrometastasis (> 2 mm), micrometastasis (0.2–2.0 mm), and isolated tumor cells (< 0.2 mm), according to the TNM staging system.

For patients with no more than 2 LN macrometastases (LN-macro), the decision to perform further axillary dissection was dependent on the operation type (breast-conserving therapy or mastectomy) and individual pathological characteristics.

### Outcomes

LymphPET accuracy was evaluated by separate comparison of the results with the final histology findings. The results were classified as true positive (TP), true negative (TN), false-positive (FP), or false-negative (FN). The evaluation of the results was based on the calculation of sensitivity [TP/(TP+FN)], specificity [TN/(TN+FP)], and NPV [TN/(TN+FN)]. The primary outcome was the NPV of LymphPET to detect macrometastases of LNs, which was defined as the proportion of nonmacrometastases of LNs in patients with negative LymphPET results.

### Statistical analysis

On the basis of the NPV for LymphPET of 87.5%, a two-sided type I error of 2.5%, and a power of 80%, we determined that 196 patients were necessary for the present study. Assuming that 3% of patients would be lost to follow up, we determined that a total of 202 patients would be needed in the first stage. For categorical variables, the χ^2^-test was used to evaluate differences, or Fisher’s exact test was used when necessary. Statistical analysis was performed in IBM SPSS version 20.0 software, and statistical significance was defined as two-sided *P* < 0.05.

## Results

### Patients

**Figure 1:**
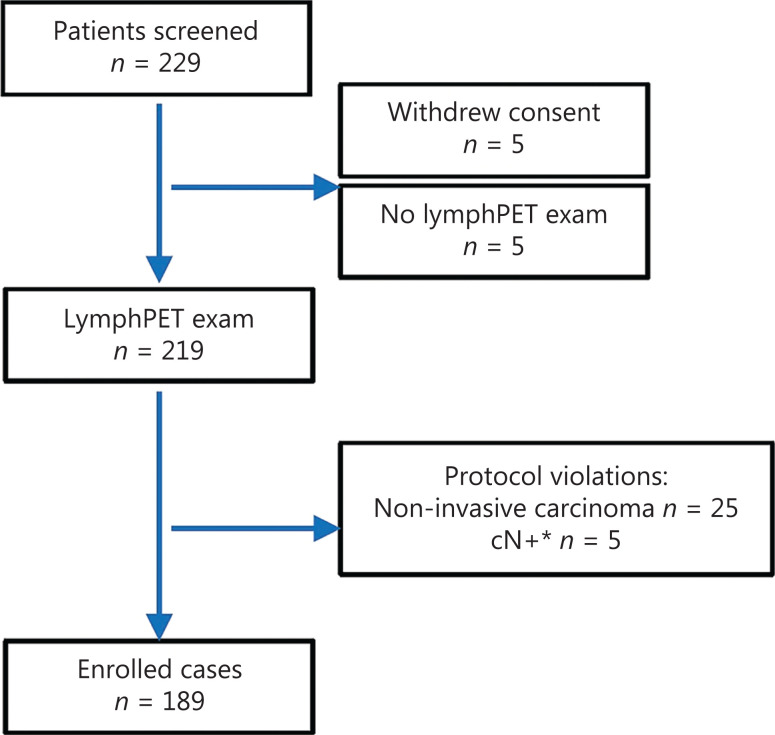
CONSORT diagram of patient disposition. *cN+: patients with a positive axillary physical examination.

From September 9, 2019, to May 30, 2020, 224 patients were screened, and 189 patients with invasive breast cancer (180 invasive ductal carcinoma and 9 invasive lobular carcinomas) underwent LymphPET followed by surgery with definitive pathological reports (**Figure 1**). The median age was 50 years. In total, 36% of patients underwent breast-conserving surgery, and 91.2% underwent SLNB. Among the patients, 53% had T1-stage disease, 64% had grade I or II disease, and 58.7% had a Ki67 index < 30%. Among the breast cancers, 63% were luminal type, 26% were HER2-positive, and 10% were triple negative. According to the pathologic report, 40 patients had at least one LN-macro, and 16 only had LN micrometastasis (LN-micro) (**Table 1**).

**Table 1:** Patient demographics and clinical characteristics

Characteristics	*n* (%)
Age (years)	49.9 (28–75)
<50	97 (51.3)
≥50	92 (48.7)
Operation type	
Breast-conserving surgery	68 (36)
Mastectomy	121 (64)
Axillary evaluation	
SLNB	133 (70.4)
SLNB, then AD	40 (21.2)
AD	16 (8.4)
Pathologic node stage	
N0	133 (70.3)
N1mic	16 (8.5)
N1	32 (17)
N2	7 (3.7)
N3	1 (0.5)
T stage	
T1a	19 (10.1)
T1b	11 (5.8)
T1c	64 (33.9)
T2	89 (47.1)
Unknown	6 (3.1)
Histology	
IDC	180 (95.2)
ILC	9 (4.8)
Grade	
I	25 (13.2)
II	96 (50.8)
III	68 (36)
Subtype	
HR positive and HER2 negative	120 (63.5)
HR positive and HER2 positive	23 (12.2)
HR negative and HER2 positive	27 (14.3)
HR negative and HER2 negative	19 (10)
Ki67 ≥30%+	78 (41.3)
LVI positive	61 (32.3)

AD, axillary dissection; HER2, human epidermal growth factor receptor 2; HR, hormone receptor; IDC, invasive ductal carcinoma; ILC, invasive lobular carcinoma; LVI, lymphovascular invasion; SLNB, sentinel lymph node biopsy.

### Efficacy outcomes

According to the ultrasound reports, 124 patients had LNs with no detected metastases (US-neg), 46 had LNs detected (US-det), and 16 had suspected metastatic LNs detected (US-met). Of the patients who had US-neg, 17 had pathologic LN-macro, and 12 had pathologic LN-micro. According to the LymphPET reports, 131 patients were LymphPET negative (maxLUV < 0.27). Of these patients, 16 had pathologic LN-macro, and 11 had pathologic LN-micro. In 58 patients who were LymphPET positive (maxLUV > 0.27), 24 had pathologic LN-macro, and 5 had pathologic LN-micro. The TP, TN, FP, FN, sensitivity, specificity, positive predictive value, negative predictive value, and accuracy for the detection of LN-macro were 23, 107, 42, 17, 57.5%, 71.8%, 35.4%, 86.3%, and 68.8% for US-neg, and 24, 115, 34, 16, 60%, 77.2%, 41.4%, 87.8%, and 73.5% for LymphPET. When clinical axillary evaluation was combined with ultrasound and LymphPET, 100 patients were found to be both LymphPET-negative and ultrasound-negative. Of these patients, 9 had LN-macro, and the NPV was 91% (**Table 2**).

**Table 2:** Diagnostic performance of LymphPET and ultrasound in axillary staging

Axillary imaging assessment	*n*	Macrometastases	Micrometastases	Non-metastases	Sensitivity	Specificity	NPV
Ultrasound‡		40	16				
US-neg	124	17	12	95	57.5	71.8	86.3
US-det	46	14	4	28			
US-met	19	9	0	10			
LymphPET†							
Negative	131	16	11	104	60	77.2	87.8
Positive	58	24	5	29			
Combination*							
Negative	100	9	9	82	77.5	61.1	91
Positive	89	31	7	51			

‡US-neg, no lymph nodes detected by ultrasound; US-det, lymph nodes detected by ultrasound; US-met, suspected metastatic lymph nodes detected by ultrasound.

†maxLUV of LymphPET was set at 0.27 (negative < 0.27, positive ≥0.27).

*Negative, no lymph nodes detected by ultrasound and maxLUV in LymphPET < 0.27; positive, lymph nodes detected by ultrasound and/or maxLUV in LymphPET ≥0.27.

Sensitivity, specificity, and NVP were calculated according to lymph node macrometastases.

The clinical characteristics of the 9 patients with FN evaluation by ultrasound and LymphPET are listed in **Table 3**. Seven patients had N1 disease, 2 had N2 disease, 1 had invasive lobular carcinoma, and 6 had lymphovascular invasion. Three patients had luminal A (patient ID: 83, 116, 129), 3 had luminal B (ID: 117, 128, 144), 2 had HER2-positive (ID: 57, 76), and one had triple-negative (ID: 147) breast cancer.

**Table 3:** Clinical characteristics of patients with a false-negative axillary node evaluation by LymphPET and ultrasound

Patient ID	Age (years)	Side	Operation	MG	Location	Size cm	Histology	Grade	LV	ER%	PR%	HER2	Ki67	No SLN removed	No. lymph node macrometastases	AD
57	44	Right	M	Mass	Center	1.5	IDC	III	+	80	60	+	15	4	1	0/19
76	67	Right	BCS	MC	Outer upper	3.7	IDC	II	–	80	10	+	20	3	1	0/12
83	41	Right	M	MC	Outer upper	1.5	IDC	II	+	80	80	–	15	5	2	0/15
116	67	Right	M	MC	Outer upper	2.8	IDC	II	+	80	80	–	10	6	3	0/12
117	41	Right	BCS	Mass	Outer upper	3	IDC	II	+	80	80	–	30	3	2	2/20
128	69	Right	M	Mass	Inner upper	2	ILC	II	–	80	20	–	30	3	2	6/24
129	47	Left	M	Mass	Outer	2.2	IDC	II	+	80	80	–	10	4	2	0/25
144	57	Left	M	Mass	Outer lower	3.5	IDC	II	+	80	80	–	30	3	1	1/22
147	61	Right	M	Mass	Outer upper	1.2	IDC	II	–	0	0	–	80	4	1	0/8

AD, axillary dissection; BCS, breast-conserving surgery; ER, estrogen receptor; PR, progesterone receptor; HER2, human epidermal growth factor receptor 2; IDC, invasive ductal carcinoma; ILC, invasive lobular carcinoma; LV, lymphovascular invasion; M, mastectomy; MC, mass with calcification; MG, mammography.

### Safety outcomes

After a median follow-up of 14 months, no adverse effects associated with the LymphPET scan were reported.

## Discussion

The SOAPET study is the first prospective trial to investigate the accuracy of LymphPET in evaluating axillary status in cN0 patients identified by clinical examination. The primary endpoint of an NPV of 87.8% for LymphPET was met. According to our results, in patients with a negative axillary physical examination, approximately 21% had LN-macro after SLNB. Even when LymphPET was combined with preoperative ultrasound, 13.7% of patients with no LNs detected by preoperative ultrasound still had LN-macro. Importantly, after combining ultrasound and LymphPET, we were able to screen potential patients who might avoid axillary evaluation, with an NPV > 90%.

Currently, in primary cN0 breast cancer, SLNB is the gold standard for regional axillary staging, providing better physical function of the upper limb than AD; however, it can also lead to specific axillary morbidity in both the early and late postoperative periods. In patients treated with SLNB alone, the rate of wound infections, axillary seromas, and paresthesia was 25% in Z0011^11^, and long-term postsurgical complications included lymphedema in 4% of patients, sensory neuropathy in 13% of patients, and motor neuropathy in 13% of patients in IBCSG 23-01^12^. Higher rates of lymphedema and an arm circumference increase > 10% have been reported after SLNB followed by axillary radiotherapy in AMAROS^13^. Therefore, screening cN0 patients to avoid surgical evaluation is an essential focus of current research. Several ongoing prospective randomized trials, such as the SOUND trial and BOOG 2013-08 trial, are comparing SLNB with observation in cN0 patients treated with breast-conserving therapy^4,14^. The key technical indicator for such trials is the NPV of preoperative assessment. A greater number of FNs indicates that LN-macros remain in the axillary, thus potentially leading to a higher local failure rate.

In initial data from the SOUND trial, 13.4% of patients in the SLNB group had LN-macro^15^, and the NPV of preoperative axillary assessment by physical examination and ultrasound was approximately 87%. Whether 13% of FN with no LN surgical evaluation might influence the outcomes remains unclear. Our study obtained a similar finding: among patients with a negative axillary physical examination and no LNs detected by preoperative ultrasound, 13.7% still had LN-macro. Therefore, determining the omission of SLNB by increasing the NPV of preoperative axillary assessment is essential.

Several studies have investigated axillary ultrasound in patients with early breast cancer. Retrospective data on 577 axillary ultrasounds have demonstrated that a negative axillary ultrasound generally excludes the presence of pN2–3 disease, whereas ultrasound cannot accurately differentiate between pN1 and pN2–3 disease^16^. FN is relatively high. In 118 patients with no node detected by ultrasound, 21% have been ultimately found to be node-positive^17^. Although these FN results have not significantly influenced adjuvant medical decision-making, the recurrence-free survival of patients with FN has been found to be equivalent to that of patients with pathological N0 disease^18^.

Other studies have further evaluated whether metabolic imaging technology might increase accuracy in the evaluation of axillary status in early breast cancer. In 349 patients with T1 stage disease who were preoperatively examined with ultrasound, MRI, and ^18^F-FDG PET/CT, the NVP was approximately 81.7%–82.6%, thus suggesting that no definitive modalities exist for detecting node metastasis in T1 breast cancer to replace SLNB^8^. With ^18^F-FDG PET/CT combined with ultrasound, 15% of 138 ultrasound-negative and ^18^F-FDG uptake-negative patients were found to have LN involvement^9^. Therefore, despite its high specificity, ^18^F-FDG PET/CT has demonstrated poor sensitivity in the detection of axillary metastases^19^. One review including 9 studies (*n* = 1,486) has reported an NPV of PET/CT for axillary staging of 77.2%^20^.

A possible explanation for this finding is that, currently, the whole-body ^18^F-FDG PET/CT system typically yields reconstructed images with a resolution of 5–15 mm, depending on the injected dose, imaging time, post-reconstruction filtering, and intrinsic resolution of the scanner, thus decreasing the system’s ability to detect small lesions (< 1 cm) and/or lesions with low tracer uptake^21^. According to our previous study, the spatial resolution of LymphPET is much higher than that of whole-body PET/CT, with 88% sensitivity and 79% specificity, and a maxLUV of 0.27 as the best cut-off value^10^. In our previous study, we developed a machine learning model integrating LymphPET and clinical characteristics for the prediction of axillary LN status in cT1-2N0-1M0 breast cancer. The performance of this integrated model showed an NPV of 96.88% in the cN0 subgroup; therefore, we believe that the use of a machine learning integrated model can greatly improve the true positive and true negative rates of identifying clinical axillary LN status in early-stage BC^22^.

In the current study, we found that the preoperative diagnostic accuracy of LymphPET was approximately equal to that of ultrasound. The sensitivity, specificity, and NPV for LymphPET were 60%, 77.2%, and 87.8%, respectively, whereas those for US-neg were 57.5%, 71.8%, and 86.3%, respectively. When LymphPET was used in the preoperative assessment of the physical examination-negative and ultrasound-negative patients, we were able to further screen 80% of patients (100/124) with a 91% NPV for axillary LN macrometastases, thus providing technical support for future studies assessing the omission of axillary evaluation.

In general, an FN rate of 10% for SLNB is acceptable. In Z0011, patients who underwent AD had 27% non-SLN positivity, on the basis of the assumption that patients underwent SLNB without AD had approximately 30% positive axillary LNs remaining.With the additional local treatment of radiotherapy, the local regional recurrence was less than 1%^11^. Similarly, in the AD group in AMAROS, 33% of patients had additional positive nodes, and the 5-year axillary LN recurrence rate was 1.19% in the SLNB group after axillary radiotherapy.^13^ Therefore, we believe that LymphPET may serve as a reliable preoperative evaluation method, and axillary surgical evaluation may be omitted if both ultrasound and LymphPET are negative.

Our study has several limitations. First, isolated tumor cells (< 0.2 mm) and micrometastases (0.2–2 mm) were not calculated in the analysis. Currently, such small metastases are difficult to detect with currently available imaging techniques. Patients with micrometastatic tumor deposits, pN0(i+) or pN1mi, do not appear to have poorer 8-year disease-free survival or overall survival than SLN-negative patients^23^. Whether treatment recommendations for systemic therapy should consider the presence of a single micrometastatic LN identified during complete serial sectioning of sentinel node(s) remains controversial^24^. Axillary radiotherapy might decrease axillary LN recurrence, particularly in patients who have undergone breast-conserving therapy with no axillary surgical intervention^25^. Second, in patients with FN detected by ultrasound and LymphPET, adjuvant systemic therapy may be inadequate without knowledge of the definitive LN status, particularly for patients with luminal type breast cancer under 50 years of age who would benefit from chemotherapy if they are LN positive. In our study, 3 patients with HER2-positive and/or triple-negative breast cancer received systemic adjuvant therapy. For 5 patients with luminal type N1 diseases, genomic signatures currently represent important progress in the optimal selection of node-positive patients (low risk by MAMMAPRINT 70 and RS < 11 by Oncotype Dx) who could potentially gain limited benefits from the addition of chemotherapy to adjuvant endocrine therapy^26,27^. One patient with invasive lobular carcinoma N2 disease (ID 128) might receive inadequate adjuvant treatment if the axillary node status is unknown. Because of these limitations, in the second stage of SOAPET, we will select patients ready for breast-conserving therapy who are both ultrasound-negative and LymphPET-negative, and genomic signatures will be evaluated in patients with luminal type breast cancer, to assess the safety of omitting surgical axillary evaluation in such a population.

## Conclusions

In conclusion, the results of our study indicate that LymphPET can be used to identify cN0 patients, thus decreasing the FN rate of clinical LN evaluation to < 10%. The second stage of the SOAPET trial is ongoing to confirm the safety of omitting SLNB according to preoperative axillary evaluation integrating LymphPET.
